# Mucopolysaccharidosis Type I: The Importance of Early Diagnosis for Adequate Treatment

**DOI:** 10.7759/cureus.50595

**Published:** 2023-12-15

**Authors:** Rui Diogo, Luísa Diogo, Rute Serra, Joana Almeida, Alexandra Oliveira

**Affiliations:** 1 Reference Centre of Hereditary Metabolic Diseases, Member of MetabERN, Centre for Child Development, Coimbra Hospital and University Centre, Coimbra, PRT; 2 Faculty of Medicine, University Clinic of Pediatrics, University of Coimbra, Coimbra, PRT; 3 Neurodevelopment and Autism Unit, Centre for Child Development, Coimbra Hospital and University Centre, Coimbra, PRT

**Keywords:** multiorgan dysfunction, hematopoietic stem cell transplant, early recognition, lysosomal storage disorder, mucopolysaccharidosis type 1

## Abstract

Mucopolysaccharidoses are rare lysosomal storage disorders in which glycosaminoglycans accumulate in tissues, causing multiorgan dysfunction. Mucopolysaccharidosis type I is an autosomal recessive disease caused by a deficiency of the enzyme alpha-L-iduronidase, resulting in the accumulation of dermatan and heparan sulfate. Early diagnosis is crucial for early treatment and improved outcomes.

We report the case of a female child with classic clinical features who was diagnosed early which allowed hematopoietic stem cell transplantation and slowed disease progression. She presented at birth with linea alba and umbilical and inguinal hernias. Since the first months of life, she had recurrent respiratory infections. At nine months, a motor delay was noticed, and at 20 months, craniosynostosis was corrected with surgery. Coarse facial features, thoracolumbar kyphosis, and hepatomegaly prompted a urinary glycosaminoglycan study at 22 months, which showed elevated levels. Alfa-L-iduronidase activity in dried blood spot testing was low, compatible with mucopolysaccharidosis type I. Molecular testing of gene *IDUA, *performed for genetic counseling, revealed the pathogenic variants c.1205G>A (p.Trp402Ter) and c.1598C>G (p.Pro533Arg) in compound heterozygosity. At 26 months, her development quotient was average for her age. She started enzyme replacement therapy at 29 months and underwent hematopoietic stem cell transplantation at 33 months, which softened the coarse features, reduced respiratory infections, and improved hepatomegaly. However, at age five, her development quotient was 76 (mean = 100, standard deviation = 15). This intellectual impairment might have been prevented with an earlier diagnosis and treatment.

## Introduction

Mucopolysaccharidoses (MPSs) are a subgroup of inherited lysosomal storage disorders caused by the deficiency of specific lysosomal enzymes on the glycosaminoglycans (GAG) catabolism pathway. The consequent storage of GAG in tissues leads to progressive multisystemic damage [1].

Mucopolysaccharidosis type I (MPS I) is an autosomal recessive disorder caused by alpha-L-iduronidase deficiency. This enzyme catalyzes the degradation of the GAG dermatan and heparan sulphate. Therefore, pathological accumulation of both GAGs occurs in patients with MPS I, with manifestations in multiple organs. This disorder has traditionally been divided into three syndromes, namely, Hurler syndrome (severe form), Hurler-Scheie syndrome (moderate form), and Scheie syndrome (mild form). However, phenotypes are present on a spectrum of severity, no biochemical differences have been identified, and clinical findings overlap. Currently, affected individuals are better divided into severe (Hurler) and attenuated (Hurler-Scheie, Scheie) forms as this distinction influences therapeutic options [2].

MPS I is a life-threatening condition with severe disease burden and premature death. Without treatment, children presenting with the severe form usually die in the first decade of life with multisystemic disease, which also affects the brain [3]. At birth, the neonate appears healthy. Early symptoms are non-specific, such as recurrent respiratory tract infections, umbilical and inguinal hernias, thoracolumbar kyphosis, and hepatosplenomegaly. Coarse facial features may not become distinguishable until after one year of age. More specific symptoms beginning in the first year include impaired hearing and vision, progressive skeletal dysplasia, followed by neurodevelopmental delay, and cardiorespiratory disease [4]. In patients with the attenuated form, symptoms usually emerge after three years of age and range in severity and rate of progression.

Early diagnosis allows timely treatment that aims to slow disease progression and improve quality of life. Currently approved treatments for the prevention of primary manifestations include enzyme replacement therapy (ERT) and hematopoietic stem cell transplantation (HSCT). The recommended treatment for patients with the severe form is HSCT, and patients with the attenuated form are often treated with ERT alone [5].

This work presents the case of a child with severe MPS I with classic clinical features with early diagnosis that allowed HSCT and slowed disease progression.

## Case presentation

In 2017, a 15-month-old girl was referred to a neuropediatric consultation because of motor delay. She was the first child of healthy, non-consanguineous parents. The gestation was uneventful. Linea alba and umbilical and bilateral inguinal hernias were noticed at birth. Surgical correction of the right inguinal hernia was performed at two months old (the left one had spontaneous resolution). By nine months old, she was unable to sit without support, raising concerns about neurodevelopment. Since the first months of life, she had recurrent otitis media, rhinitis, and wheezing.

Clinical findings

**Figure 1:**
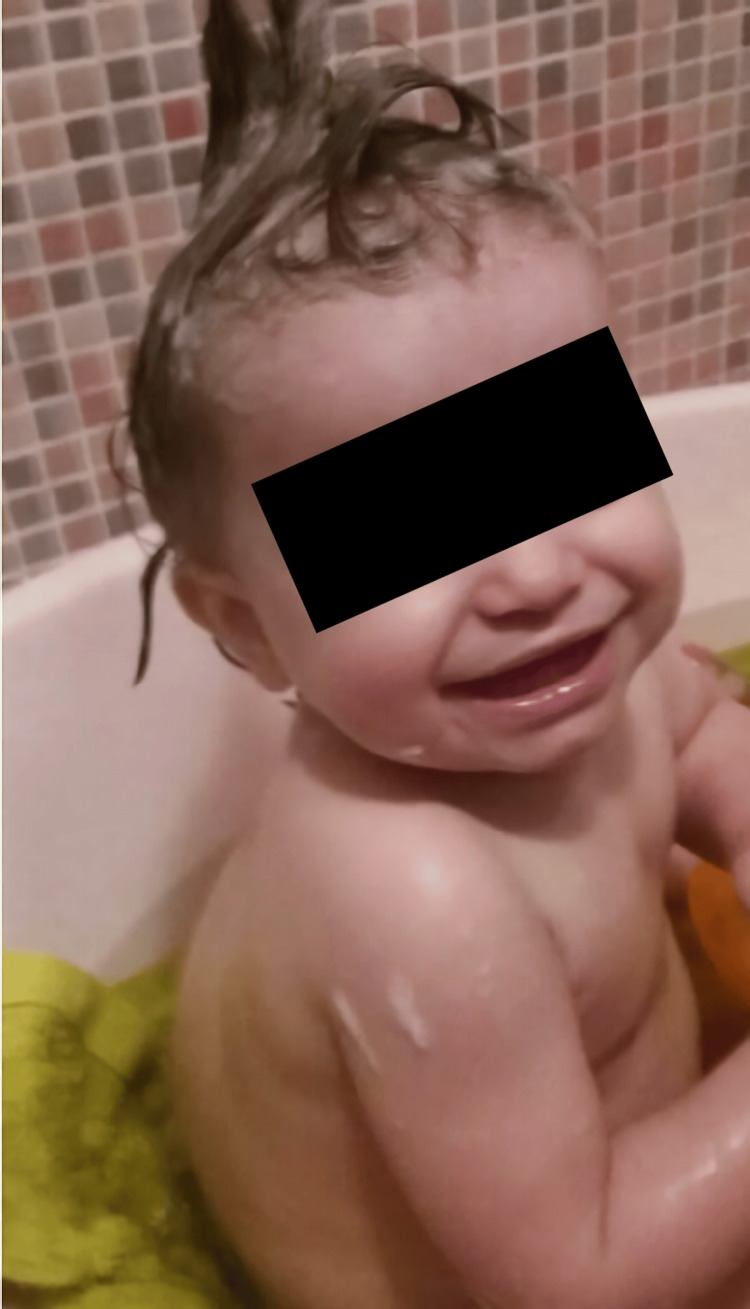
Coarse facial features and thoracolumbar kyphosis at 15 months.

Physical examination showed coarse facial features (prominent supraorbital ridges, hypertelorism, broad nose, and thick lips) (Figure 1), dolichocephaly (craniosynostosis suspicion), mild thoracolumbar kyphosis, hepatomegaly, and umbilical hernia. The remaining examination was otherwise normal.

The evolution of weight and length was in the 15th-50th percentile, and the cephalic perimeter was in the 50th-85th percentile.

**Figure 2:**
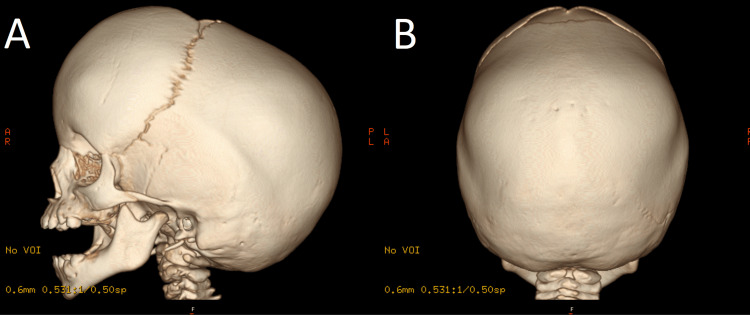
Cranial computed tomography performed at 18 months. (A) Increased anteriorposterior diameter (dolichocephaly). Also, left temporoparietal suture has decreased patency. (B) Sagittal and lambdoid sutures are closed bilaterally.

At 18 months, a computed cranial tomography showed a sagittal and lambdoid craniosynostosis, with increased anterior-posterior diameter (Figure 2). At 20 months, she underwent a cranial vault remodeling.

Diagnostic assessment

MPS was suspected at 22 months old, and the urinary GAG study showed elevated levels at 308.2 mg/L (normal range = 39.5-51.5 mg/L).

The child was then referred to Genetic and Inherited Metabolic Disorders consultations. Alfa-L-iduronidase activity in dried blood spot testing (FIND Project [6]) was low: 0.01 nmol/h/spot (normal range = 0.1-0.9 nmol/h/spot), suggestive of MPS I.

Molecular testing of the gene *IDUA *revealed the nonsense pathogenic variant c.1205G>A (p.Trp402Ter) in exon 9 and the missense pathogenic variant c.1598C>G (p.Pro533Arg) in exon 11, in compound heterozygosity.

**Figure 3:**
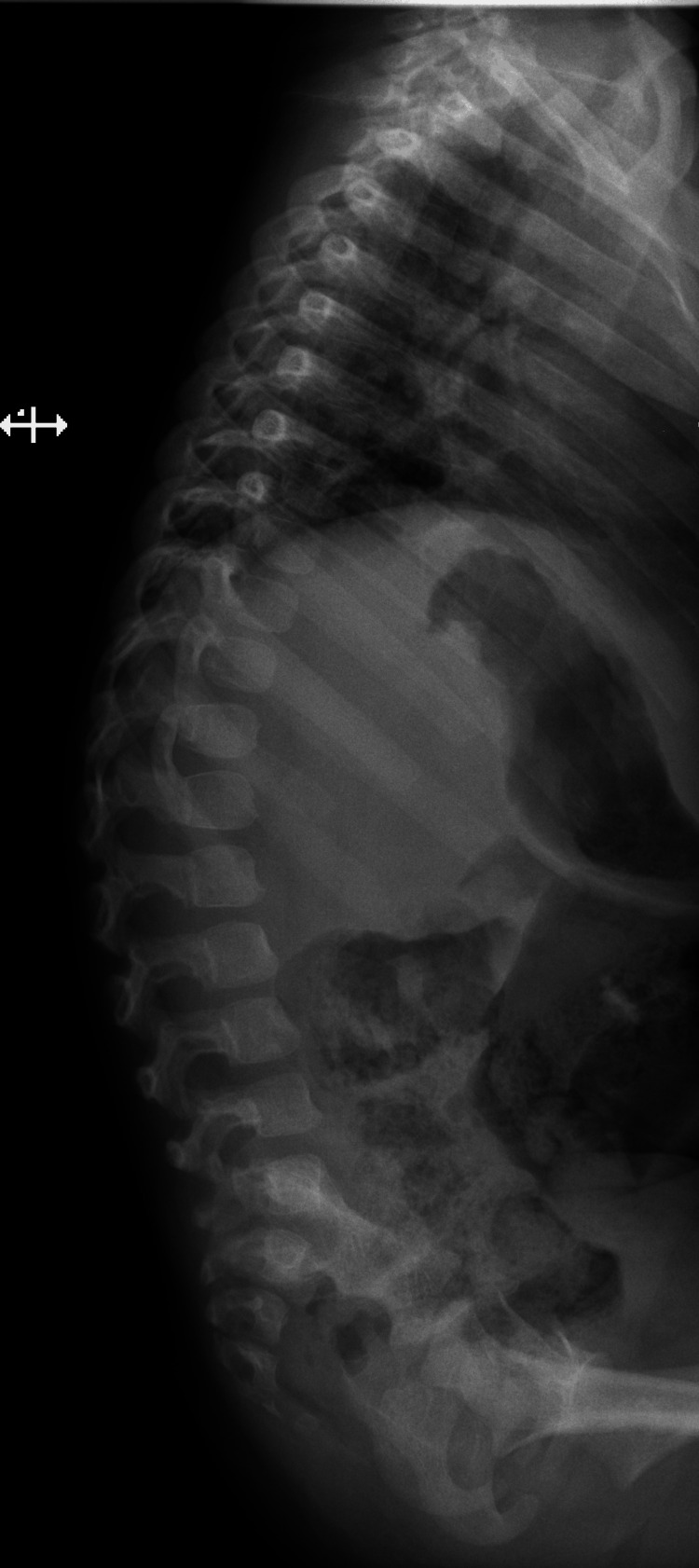
Spine radiography at 27 months (lateral view). Absence of natural spine curves, with a mid-thoracolumbar kyphosis (mostly positional).

Further evaluations at diagnosis and during evolution were performed according to MPS guidelines [5] and are presented in Table 1. Figure 3 shows spine radiography performed at 27 months.

**Table 1:** Clinical manifestations, complementary evaluations, and treatment during follow-up. According to mucopolysaccharidosis type I guidelines for management and treatment [5], patients should receive a comprehensive baseline evaluation, including neurologic, ophthalmologic, auditory, cardiac, respiratory, gastrointestinal, and musculoskeletal assessments, with monitoring every 6-12 months, by individualized specialty assessments.

Consultation	Clinical manifestations and treatment
Pneumology	Recurrent rhinitis and wheezing, snoring – inhaled and nasal corticosteroids
Cardiology	No symptoms – normal electrocardiography; echocardiogram: dysplastic mitral valve with moderate insufficiency and moderate aortic insufficiency – medicated with lisinopril
Surgery	Mild umbilical hernia – corrective surgery at 40 months
Neurosurgery	No need for further neurosurgeries
Orthopedics	Bilateral carpal tunnel syndrome – corrective surgery at the age of six
Ophthalmology	No symptoms – mild right corneal opacity, normal fundus evaluation, and normal intraocular pressure
Otorhinolaryngology	Recurrent rhinitis, otitis media (normal audiometry and tympanogram) – myringotomy at 28 months and adenotonsillectomy at 30 months old, repeated adenoidectomy and myringotomy at the age of seven due to chronic suppurative otitis
Exam evaluations	
Abdominal echography (28 months, 32 months and 44 months)	At 28 months, mild hepatomegaly, right lobe with 120 mm, inferior vena cava plane 120 mm, and mild splenomegaly with 88 mm. At 44 months, the right lobe was 101 mm, and the left lobe was 81 mm, with the spleen measuring 71 mm
Musculoskeletal radiography (multiple from 15 months)	Incomplete fusion of C4 vertebra, beaking of thoracolumbar vertebra bodies, dorsolumbar kyphoscoliosis (Figure 3), right acetabular dysplasia, distal femur diaphysis widening
Cranial magnetic resonance imaging (27 months)	Enlarged perivascular spaces in the peri trigonal regions, with hyperintense signal in DP, T2, and fluid-attenuated inversion recovery, of the white substance, involving periventricular regions, corona radiata, external and subcortical capsules, that can be secondary to gliosis, demyelination or dysmyelination. J-shaped sella turcica
Spine magnetic resonance imaging (27 months and seven years old)	Odontoid hypoplasia, mild periodontal ligament thickening, reduction of the subarachnoid space in this location
Polysomnography (32 months)	Moderate obstructive sleep apnea syndrome
Thoracic computed tomography (seven years old)	Bronchi walls parietal thickening with inferior lobe predominance. Bilateral densification of medium lobe and lingula, compatible with atelectases

**Figure 4:**
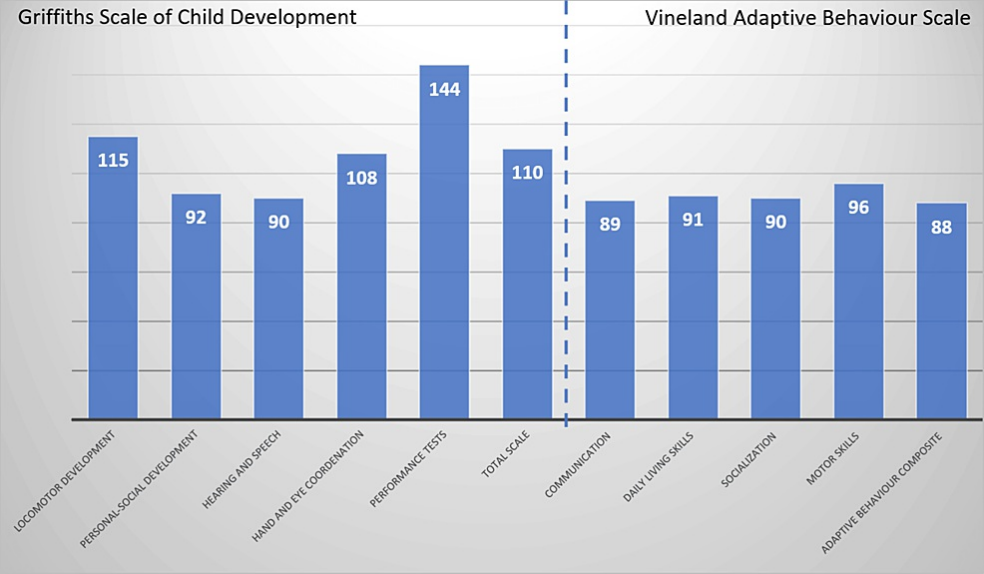
Griffiths Scale of Child Development and Vineland Adaptive Behaviour Scale at 26 months old (medium 100 = standard deviation = 15).

Neurodevelopment evaluation with Griffiths Mental Development Scale and adaptive behavior with Vineland Adaptive Behaviour Scale at 26 months old was average for age (Figure 4).

Therapeutic intervention

**Figure 5:**
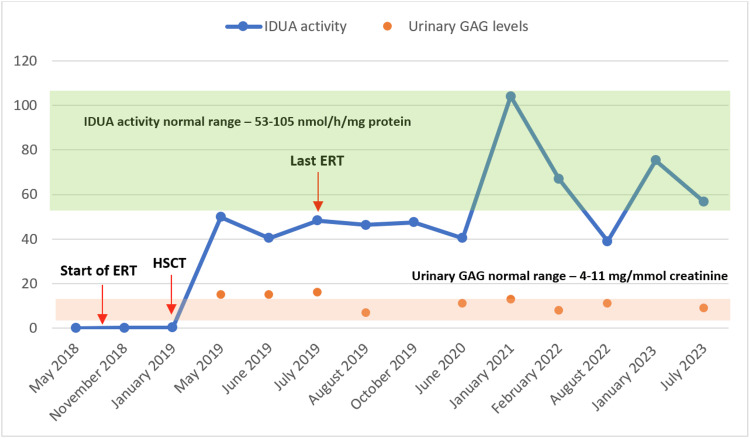
Evolution of alfa-L-iduronidase (IDUA) activity in leucocytes and urinary glycosaminoglycans (GAG). IDUA activity normal range levels are 53-105 nmol/h/mg protein (y axis). Urinary GAG normal range levels are 4-11 mg/mmol creatinine (y axis).

The child started intravenous ERT with laronidase (Aldurazyme®) at 100 U/kg/dose weekly at 29 months old. Additionally, she was proposed for HSCT, which occurred at 33 months old. Full-donor chimerism was achieved four months after HSCT. She maintained ERT weekly until six months after the HSCT. The evolution of alfa-L-iduronidase activity levels and urinary GAG is shown in Figure 5.

**Figure 6:**
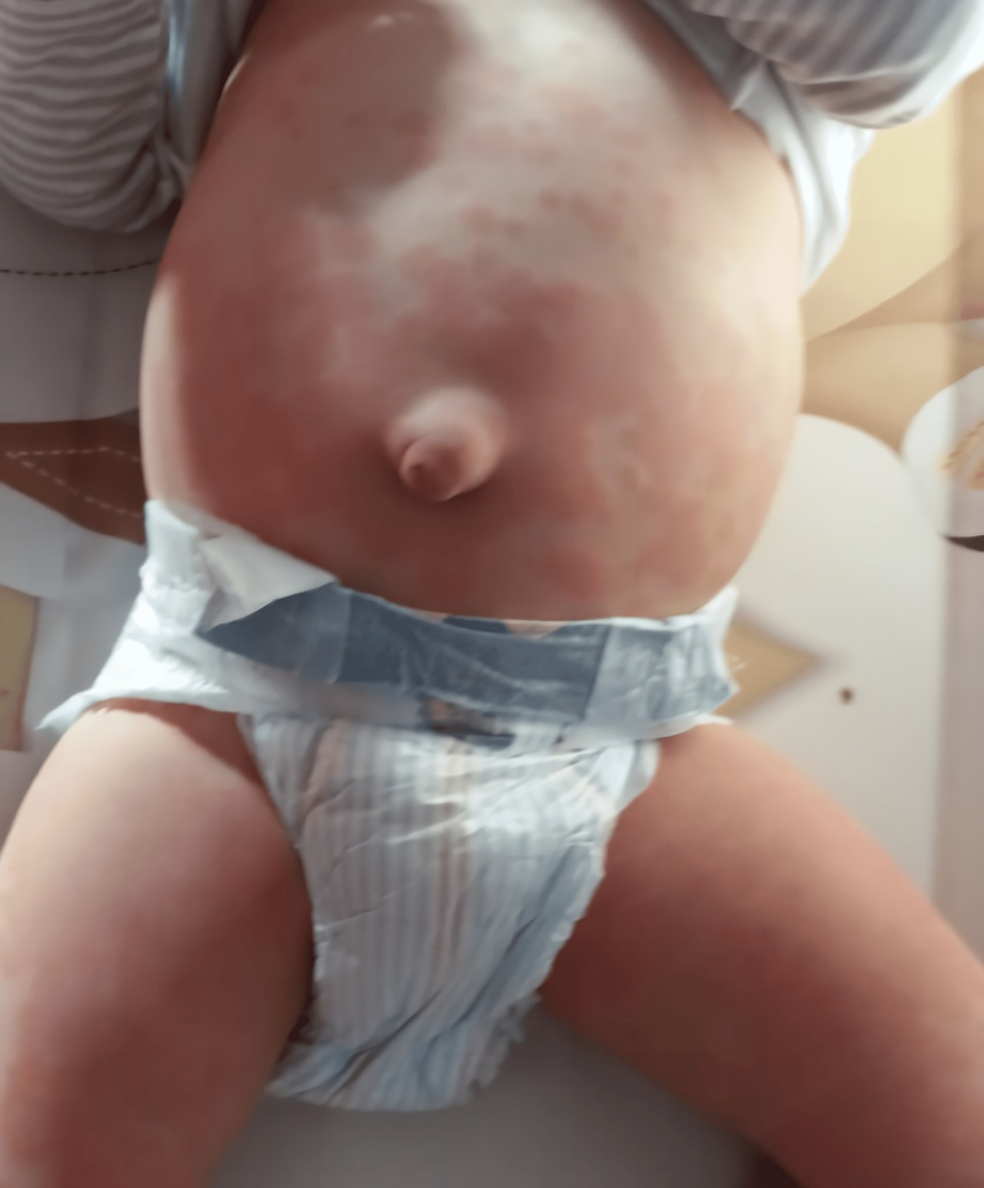
Cutaneous graft-versus-host disease. An umbilical hernia can also be noticed.

The main complications after HSCT were grade II graft-versus-host disease (cutaneous involvement, Figure 6), autoimmune hemolytic anemia, reinfections of cytomegalovirus and Epstein-Barr virus, hypogammaglobinaemia secondary to immunosuppression, and iron deficiency. All resolved with standard therapy (corticoids, immunoglobulin, antivirals, monoclonal antibody, and iron supplementation).

**Figure 7:**
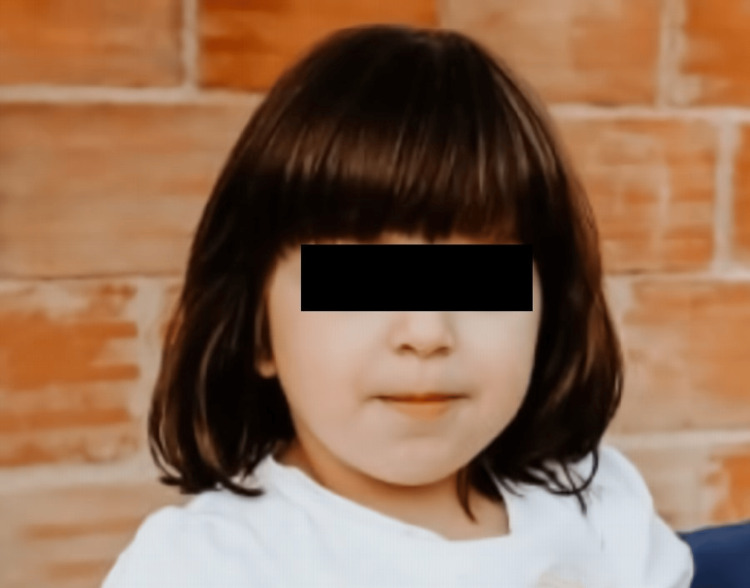
Photograph at age five showing softening of coarse features.

**Figure 8:**
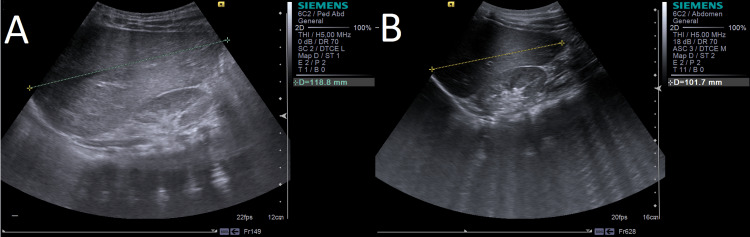
Liver ultrasound at 28 months (A) and 44 months (B). (A) Hepatomegaly at 28 months, with right lobe sizing 118.8 mm. (B) Right lobe with 101.7 mm, showing liver size reduction at 44 months.

Currently, she is seven years old, maintains follow-up by a multidisciplinary team, and is clinically stable. The coarse features softened (Figure 7), and hepatomegaly improved (Figure 8). Although upper airway infections reduced in number, she was submitted to adenoidectomy and myringotomy at the age of seven due to chronic suppurative otitis (Table 1).

**Figure 9:**
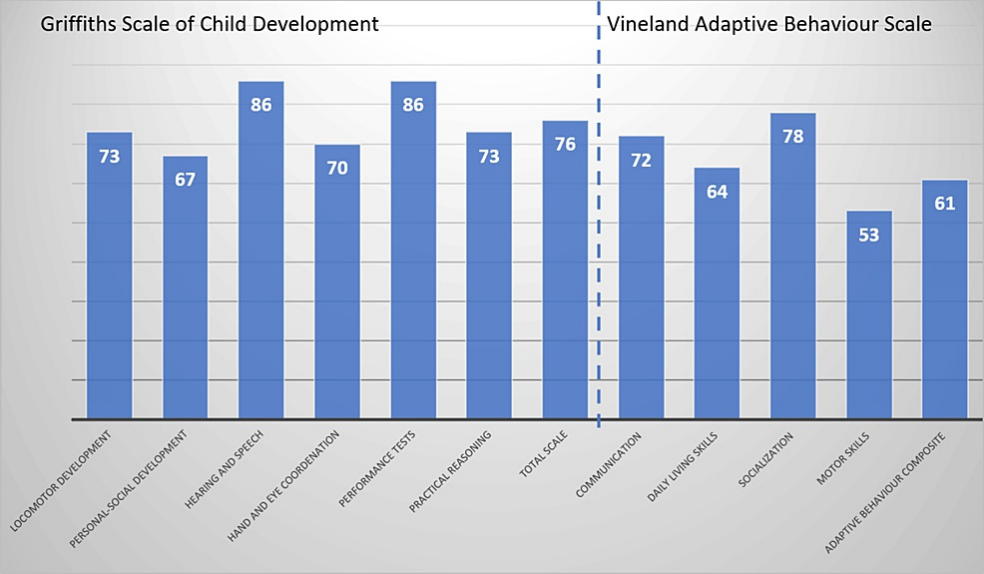
Griffiths Scale of Child Development and Vineland Adaptive Behaviour Scale at 60 months old (medium = 100, standard deviation = 15).

The last neurodevelopment evaluation at five years of age showed a Griffiths general quotient of 76 (average = 100, standard deviation = 15) and a Vineland’s adaptative behavior composite score of 61 (average = 100, standard deviation = 15) (Figure 9). She is in regular school with education support.

## Discussion

MPS I has an estimated prevalence of 0.69 to 3.8 per 100,000 live births [7]. At birth, the neonate can look healthy. Early symptoms include progressive coarse facial features, recurrent rhinitis, upper air obstruction, abdominal or inguinal hernias, thoracolumbar kyphosis, and hepatosplenomegaly. Upper air obstruction can result in sleep apnea and respiratory tract and ear infections [8]. Cardiac involvement includes thickened cardiac valves, which can lead to poor mobility, regurgitation, or stenosis [9]. Dysostosis multiplex is also one of the more common manifestations [10]. Spine involvement includes abnormalities affecting the intervertebral discs, vertebrae, odontoid process, and dura, causing spinal deformity and cord compression [11]. Central nervous system involvement can include white matter injury, enlargement of perivascular spaces, hydrocephalus, brain atrophy, characteristic enlargement of the subarachnoid spaces, and compressive myelopathy, among other manifestations [12]. Neurodevelopment can become impaired in the first year of life, with progressive decline after the second year [13]. Ophthalmic manifestations include corneal clouding, ocular hypertension/glaucoma, retinal degeneration, optic nerve swelling/atrophy, refractive errors, and ocular motility abnormalities [14,15].

The child described in this report presented with typical clinical manifestations, namely, multiple hernias, recurrent otitis media, rhinitis, wheezing, coarse facial features, craniosynostosis, thoracolumbar kyphosis, hepatomegaly, dysplastic mitral valve with insufficiency, aortic insufficiency, and mild corneal opacity. These features led to MPS suspicion before two years of age.

Urinary GAG analysis should be conducted upon suspicion as a fast and efficient method to screen for the disease. Enzyme testing (measurement of alfa-L-iduronidase activity) is the gold standard for diagnosis and, in HSCT-treated patients, is paired with urinary GAG for treatment efficacy monitoring [1]. Molecular diagnosis is the confirmatory test. The alpha-I-iduronidase gene has been mapped to chromosome 4p16.3. The c.1205G>A (p.Trp402Ter), c.208C>T (p.Gln70Ter), and c.1598C>G (p.Pro533Arg) mutations are mainly responsible for the disease in the European population [16-18]. Our patient’s genetic study identified two of the most common mutations in Europe.

According to the European consensus (2011) [19], the preferred treatment strategy is HSCT for patients with MPS diagnosed before 2.5 years, with a development quotient over 70.

ERT, which must be administrated intravenously every week, is ineffective in preventing neurocognitive and behavioral impairment as the recombinant enzyme does not cross the blood-brain barrier. Nevertheless, it remains the best option for late-presenting or late-diagnosed patients as it improves some of the life-threatening manifestations, such as respiratory impairment. Although it is a high-risk procedure, with non-negligible mortality and morbidity, early HSCT may prevent cognitive decline and improve other clinical manifestations of the disease with a better quality of life in the long term.

However, how early is early enough? On retrospective analysis of our patient’s history, we can conclude that it took too long before the MPS I diagnosis and treatment were assumed: from 15 months of age to 29 months (ERT) and 33 months (HSCT). She was transplanted over the recommended age limit, according to European Guidelines [19]. Although neurologic disease progression is assumedly better after HSCT than on ERT alone, she presented some intellectual and adaptive impairment, which might have been prevented with an earlier diagnosis. Training of healthcare professionals and dissemination among the public are needed to valorize the first signs of the disease as when the complete picture is present, diagnosis is easy but late.

Neonatal screening of MPS I is being advocated based on the knowledge that GAG accumulation is prenatal and progressive and on the improved prognosis of MPS I younger siblings submitted to earlier treatment [20]. Presently, it is not included in the Portuguese Newborn Screening Program.

Currently, the only way to treat MPS I patients adequately is through improving MPS awareness among healthcare professionals.

## Conclusions

MPS I is an inherited autosomal recessive disorder with a multisystemic (including neurological) progressive course. Clinicians may suspect this diagnosis in children with coarse features, abdominal/inguinal hernias, recurrent upper respiratory infections, or dorsolumbar kyphosis. The Portuguese FIND Project provides physicians easy and quick access to MPS I enzyme diagnosis in a dried blood spot sample. Early diagnosis and treatment with HSCT can slow disease progression and improve quality of life. In this context, neonatal screening for MPS I may be helpful.
